# The predictive value of intravesical prostatic protrusion on the outcome of trial without catheter in patients with acute urinary retention from benign prostatic hyperplasia at Jos University Teaching Hospital, Nigeria: a prospective observational study

**DOI:** 10.11604/pamj.2022.42.246.30685

**Published:** 2022-08-02

**Authors:** Ayodele Olufikayo Oshagbemi, Chimaobi Gideon Ofoha, Idorenyin Cletus Akpayak, Samaila Ibrahim Shuaibu, Nuhu Kutan Dakum, Venyir Mamzhi Ramyil

**Affiliations:** 1 Department of Surgery, Jos University Teaching Hospital (JUTH), Plateau State, Nigeria,; 2 Department of Surgery, College of Medicine, Jos University Teaching Hospital, Plateau State, Nigeria

**Keywords:** Value, intravesical, prostatic, protrusion, outcome

## Abstract

**Introduction:**

acute urinary retention represents a significant and painful event in the natural history of benign prostatic hyperplasia. This study was to determine the value of intravesical prostatic protrusion in predicting the outcome of trial without catheter in patients presenting with acute urinary retention from benign prostatic hyperplasia.

**Methods:**

this was a prospective observational study carried out over a one-year period among 78 patients with acute urinary retention from benign prostatic hyperplasia who presented at the Accident and Emergency Department of Jos University Teaching Hospital. They were clinically evaluated, and a urethral catheter was passed to relieve the retention. Trans-abdominal ultrasound assessment of intravesical prostatic protrusion, was performed after relief of acute urinary retention. Patients were placed on tamsulosin tablets 0.4mg daily for three days and they had a trial without catheter on the third day. A receiver operating characteristic curve was used to determine the predictive power of intravesical prostatic protrusion on the outcome of trial without catheter in patients with acute urinary retention from benign prostatic hyperplasia. A p value of <0.05 was considered as significant.

**Results:**

seventy-eight patients were enrolled in the study. The mean age and was 65.00 (SD 7.28) years. The mean intravesical prostatic protrusion, voided volume and maximum flow rate were 13.04 (SD 10.94) mm, 89.46 (SD 6.14) mls and 7.63 (SD 5.69) ml/s respectively. Intravesical prostatic protrusion (area under the curve= 0.843, p=0.001) predicted the outcome of trial without catheter with a cut off mark of <7.4, using the receiver operating characteristic curve.

**Conclusion:**

intravesical prostatic protrusion significantly predicted the outcome of trial without catheter in patients with benign prostatic hyperplasia presenting with acute urinary retention. It is a useful tool in the initial evaluation of patients with benign prostatic hyperplasia presenting with acute urinary retention.

## Introduction

Acute urinary retention (AUR) is a common urological complication that presents with a sudden inability to void voluntarily, associated lower abdominal pain, with relief of symptoms following catheterization [1]. It is one of the significant and most serious complications of benign prostatic hyperplasia (BPH) [2,3]. Benign prostatic hyperplasia is a major health problem for aging male and has an adverse effect on a patient´s quality of life [4]. Studies have estimated the frequency of AUR to be about 5-25 per 1000 person-years or 0.5% to 2.5% per year [5,6]. Management of AUR consists of immediate bladder decompression by urethral catheterization, which is usually followed by an early BPH-related surgery [7,8]. Prolonged urethral catheterization has been associated with impaired quality of life due to catheter associated urinary tract infection, discomfort and non-deflating catheter balloon [9].

The presence of the urethral catheter results in bacterial colonization of the bladder at a rate of 4% per day increasing the risk of peri-operative sepsis [10]. Prostatectomy on patients with AUR was found to be associated with increased morbidity and an increased risk of death during and after surgery [11]. Alpha-blockers (e.g. doxazosin or tamsulosin) reduces functional symptoms of BPH [3,12], thereby improving flow rates and bladder emptying, and it is thought that they help to reduce bladder outlet resistance [13]. Trial without catheter (TWOC) is a therapeutic method to induce self-voiding after a certain period of urethral catheterization and it is being attempted in patients with AUR [8]. A successful TWOC may lead to the avoidance of prostatectomy in 23% of patients presenting with AUR [14].

Intravesical prostatic protrusion (IPP) is the degree of protrusion of the prostate into the urinary bladder seen on ultrasound scan. It has been shown to correlate well with predicting bladder outlet obstruction in patients with BPH [5]. IPP causes a “ball-valve” type of obstruction. IPP is measured from the protruding prostate tip perpendicularly down to the bladder circumference at prostate base taken at mid- sagittal view. Grade 1 is assigned if the protruding tip is less than 5mm (< 5mm), grade 2 when the tip is 5 mm to 10 mm (5mm-10mm) and grade 3 when the tip is more than 10 mm (>10mm) [12]. Grade 3 IPP is also associated with a higher bladder outlet obstruction index than grade 1 and 2 patients with BPH [13]. Sharis *et al*. [5] in Malaysia, found that patients with grade 1 prostate may benefit from TWOC. However, patients with grade 3 prostate were less likely to benefit from TWOC and would require a more definitive surgical procedure. This was similar to prospective studies by Mariappan *et al*. [15] in Edinburgh, and Tan *et al*. [16] in Singapore in which IPP was found to predict the outcome of TWOC following AUR.

The aim of this study was to find out the predictive value of IPP on the outcome of TWOC in patients presenting with AUR from BPH in Jos, Nigeria.

## Methods

**Study design and setting:** this study was a prospective observational study over a period of one year. It was carried out at the Jos University Teaching Hospital which is a tertiary health institution located in Plateau State Capital, Jos, Nigeria.

**Study population:** the subjects were patients presenting to the accident and emergency department with first episode of AUR attributable to BPH who consented for this study subjects were recruited via a non-probability (purposive) sampling technique. Those with AUR who had been receiving treatment for BPH, patients with gross haematuria, urinary tract infection and carcinoma of the prostate were excluded from the study. Patients with failed urethral catheterization and those with acute on chronic urinary retention with drainage of greater than 1000 mls of urine after relief of retention [17] were also excluded from the study. The projected sample size that is required to meet the set objectives at ninety-five percent (95%) confidence level was calculated using Fischer´s formula [18]:


$$n=\frac{Z^{2}pq}{\partial^{2}}$$

Where: n = the desired sample size; Z = the standard normal deviation corresponding to 95% level of confidence. The value obtained from the normal distribution is 1.96; p = the proportion of patients with acute urinary retention secondary to BPH that present at the accident and emergency of Jos University Teaching Hospital, estimated at 23% (0.23). That is, an average of 34 cases of acute urinary retention secondary to BPH out of 147 urological emergencies presenting at the accident and emergency per year (2014-2016).


q=1−p=1−0.23=0.77

∂ = degree of accuracy desired (i.e. precision) is set at 10% (0.1). The sample size:


$$n=\frac{{\left(1.96\right)}^{2}\times 0.23\times 0.77}{{\left(0.1\right)}^{2}}=68.03$$

The sample size therefore approximates to 68 patients given the margin of error. With 10% attrition of 6.8, total sample will be 75 patients. However, 78 patients were recruited for the study.

**Study procedures:** each of the subjects had relief of urinary retention by passing a size 16F latex Foley urethral catheter after the benefits, risks, and complications have been explained and discussed with the patient. Equipment used for the procedure included a sterile tray, cetrimide, sterile gauze, sterile gloves, sterile drapes, sterile 2% lidocaine gel (with a blunt tip urethral applicator), sterile water, 10mL syringe and a size 16 F latex Foley urethral catheter. The procedure was carried out with an assistant, and the dignity and privacy of the patient was maintained by utilizing screens or curtains. The patient was positioned supine with flexion and external rotation of the hip, abduction of the femur and flexion of the knees. The genitalia were exposed. Having ensured hand hygiene by scrubbing, a sterile glove was worn. Cleaning of the penis using cetrimide was done, sterile drapes was used to create a sterile field around the penis. The penis was held with the non-dominant hand, 10mL of 2% lidocaine gel was instilled into the urethra and a Cunningham penile clamp was applied to prevent spillage of the anaesthetic lubricant. A waiting period of 5 minutes was observed before proceeding with the urethral catheterization.

The catheter was held by the dominant hand and a generous amount of lidocaine gel was applied. While holding the penis at approximately 90° with gentle stretching, a 2-way size 16F latex Foley catheter was introduced and advanced until the proximal Y-shaped ports was at the external meatus. After visualization of urine draining from the larger port (while the proximal port was at the level of the external meatus), the balloon was inflated by injecting 10ml of sterile water through the inflation port. A urine drainage bag was connected to the drainage port. The catheter was gently withdrawn from the urethra until resistance was met. The volume of urine drained was recorded. If the volume of drained urine was > 1000ml, the patient was presumed to have acute-on-chronic urinary retention [17] and therefore excluded from the study for reasons stated earlier. The urine drainage bag was removed and a spigot was applied to the urethral catheter after relief of AUR.

**Data collection:** instruments used for this study are; uroflowmeter (NIDHI Flow-814), which is a fully automated microprocessor based device with digitally controlled weight-based flow transducer designed to monitor the urinary volume and flow rate within a urine collection beaker during micturition. It has a microprocessor based Ad-On module that provides the statistical parameters of urine flow, a transducer, micturition chair and funnel, urine collection beaker (2000ml capacity), and an EPSON compatible matrix printer. A GE Logiq S expert 052128 model ultrasound was also used in this study.

Each subject had clinical evaluation. They were relieved of urinary retention by passing urethral catheter. Mid-stream urine specimen was collected for urine microscopy culture and sensitivity. The volume of urine drained was recorded using a graduated container and a spigot was applied to the urethral catheter after relief of AUR. Blood samples were taken for urea, electrolyte, creatinine and prostate specific antigen (PSA) test. Patients had a trans-abdominal ultrasound evaluation of the prostate and urinary tract, including IPP and prostate volume using a GE Logiq S expert 052128 model ultrasound. This was performed by the same consultant radiologist in the hospital when the bladder volume was between 100-200mls. A curvilinear probe of 3.5 MHz was used to measure the IPP along the mid sagittal view. IPP (measured in millimeters) was measured as the vertical distance in the mid sagittal image from the tip of the protruding prostate to the base of the urinary bladder [19].

The spigot was then removed and a urine drainage bag applied. The patients were placed on tamsulosin tablets 0.4mg daily and instructed to report after three days at the urology outpatient department with a fasting blood glucose (FBG) result and for TWOC. Patients were re-assessed on the third day to determine eligibility for the study. They were instructed to drink 750mls of water, and when they felt the urge to urinate a TWOC was attempted under uroflowmetric study. The maximum flow rate (Qmax), average flow rate (Qave), and voided volume were recorded in a structured proforma. A size 16F silicone Foley catheter was passed for all patients with unsuccessful TWOC and they were taken back to the urology outpatient department for further instruction before sending them home. All patients recruited for this study were given an appointment for clinic visit for further management.

**Definition:** failed TWOC: inability to pass urine, or passage of urine less than 150mls and a Qmax less than 10ml/s [20].

**Statistical analysis:** all data obtained from the study subjects were collated and subjected to statistical analysis using Statistical Package for Social Sciences (SPSS®) version 23. SPSS is a statistical software suite developed by IBM and it is based in Chicago in USA. Tables were used to express the data. Bar and pie charts were used for descriptive analysis of demographics. T-test was used for inferential analysis where appropriate. The receiver operating characteristic curve (ROC) was used to determine the predictive power of IPP on the outcome of TWOC in patients with AUR attributable to BPH. A p-value of < 0.05 was considered as statistically significant.

**Ethical consideration:** permission to conduct this study was obtained from the Research and Ethics Committee of Jos University Teaching Hospital (JUTH) with reference number JUTH/DCS/ADM/127/XXVII/798. Informed consent was also obtained from all patients who met the criteria for inclusion in the study. Only consenting patients were enrolled. Subjects were at liberty to opt out of the study at any stage without any consequence in terms of care or treatment. The results of each patient´s evaluation was treated confidentially and no financial obligations pertaining to this study was borne by the patient.

## Results

**General characteristics of the study population:** a total of 81 men who gave consent and met the inclusion criteria were recruited into the study. Three patients were excluded from the study because they did not turn up for trial without catheter, leaving 78 (96%) patients for analysis. The age range of the subjects was 52-82 years with a mean age of 65.00 (SD 7.28) years. The demographic information of the study participants is shown in Table 1.

**Table 1:** demographic variables of 78 patients who had trial without catheter

Demographic variables	Frequency	Percentage (%)
**Educational level**		
None	13	16.67
Primary	12	15.38
Secondary	23	29.49
Tertiary	30	38.46
Total	78	100.00
**Occupation**		
Retiree	31	40.00
Farming	17	22.00
Civil servants	14	18.00
Businessmen	12	15.00
Others	4	5.00
Total	78	100.00

**Figure 1:**
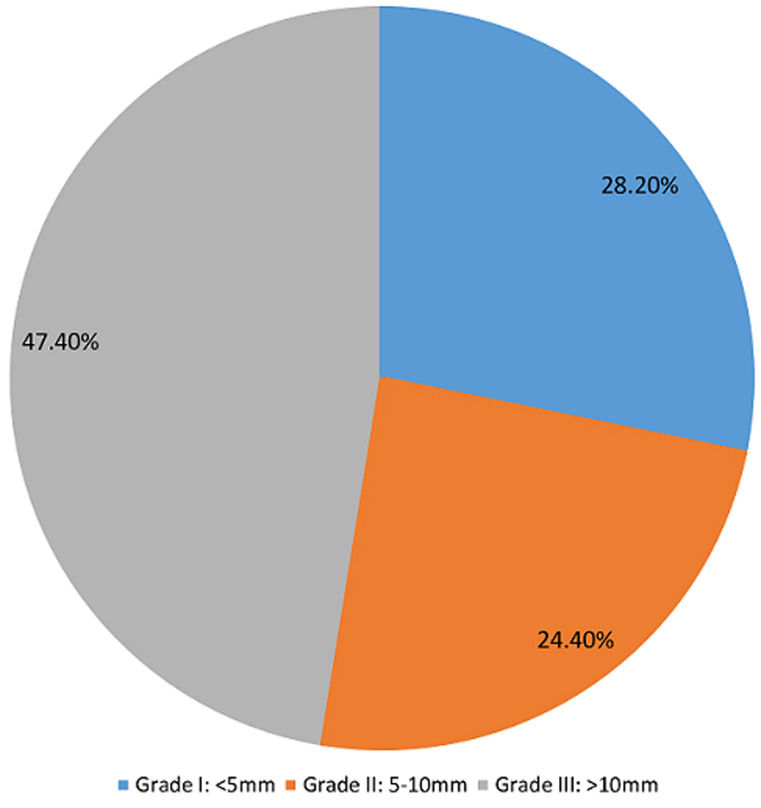
intravesical prostatic protrusion grade distribution of 78 study participants who had trial without catheter

**Figure 2:**
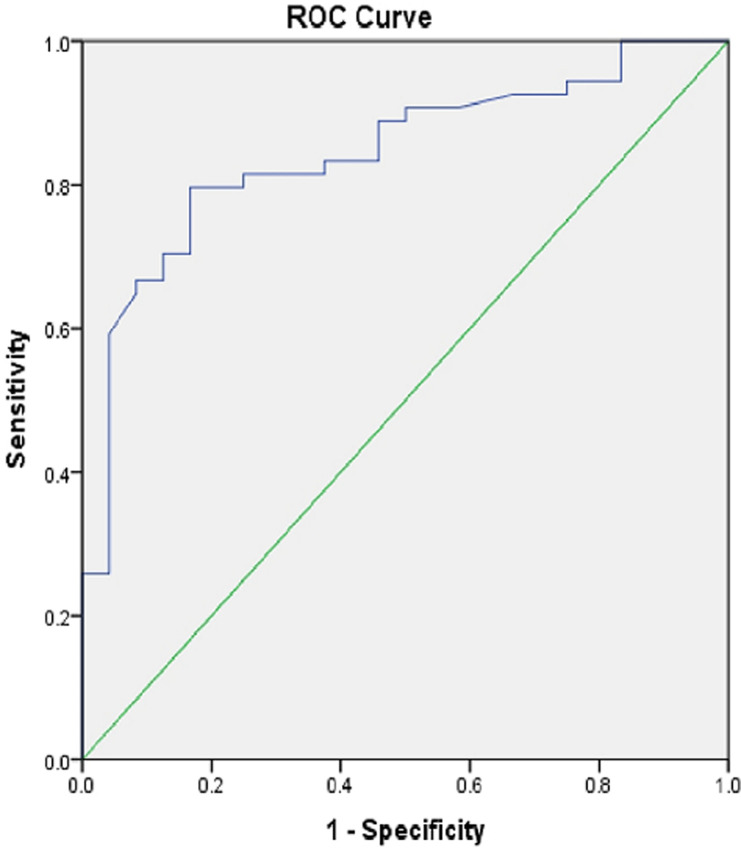
the receiver operator curve of intravesical prostatic protrusion against the outcome of trial without catheter

**Main results:** the IPP grade distribution of the study participants is shown in Figure 1. The mean IPP was 13.04 (SD 10.94) mm, while the Qmax was 7.63 (SD 5.69) ml/s. Other descriptive statistics of the study population are shown in Table 2. Twenty-four patients (30.8%) had a successful TWOC. The area under the curve (AUC) of ROC for IPP and the outcome of TWOC was 0.843 (p=0.001). The sensitivity and specificity was 79.6% and 83.3% respectively. The cut off was 7.4mm. The positive predictive value (PPV) and negative predictive value (NPV) {cut off of 7.4} were 91.5% and 64.5% respectively. The receiver operator curve of IPP against the outcome of TWOC is shown in Figure 2.

**Table 2:** descriptive statistics of 78 patients who had trial without catheter

Parameters	Mean	SD
Urine volume (mls)	708.05	130.11
Prostate volume (mls)	80.63	4.32
IPP (mm)	13.04	10.94
PSA (ng/ml)	4.82	2.12
FBG (mmol/ml)	4.54	0.77
Voided volume (mls)	89.46	6.14
Maximum flow rate (ml/s)	7.63	5.69
Average flow rate (ml/s)	4.53	3.41

IPP: intravesical prostatic protrusion; PSA: prostate specific antigen; FBG: fasting blood glucose

## Discussion

The objectives of this study were to determine the IPP, voided volume and Qmax of BPH patients presenting with AUR, and to find out the relationship between IPP and the outcome of TWOC. The mean IPP, voided volume of urine, and Qmax, were 13.04 (SD 10.94) mm, 89.46 (SD 6.14) mls and 7.63 (SD 5.69) ml/s respectively. IPP was found to predict the outcome of TWOC. The mean voided volume at uroflow and mean Qmax was 89.46ml and 7.63ml/s respectively. Mohammed *et al*. [21] reported a higher mean Qmax of 17.56ml/s, which may be due to the fact that their study was a randomized prospective study in which one group had TWOC on the third day and another group had TWOC on the seventh day post relief of AUR, as against this study in which all patients had TWOC on the third day after urethral catheterization. The low mean of Qmax in this study showed that most of the participants had a failed TWOC. The definition of success for this study was a Qmax of 10ml/sec [20]. Based on this definition of success, 24 (30.8%) of the patients were adjudged to have successful TWOC. This is similar to the study by Bansal *et al*. [22] who reported 33.7% as successful. It is also similar to the work done by Bhomi *et al*. [23] and Shanmugasundaram *et al*. [24] in which a successful TWOC was reported in 43.75% and 43.9% of patients respectively. However, this is at variance with the study by Sharis *et al*. [5] who reported a success rate of 50%. The small number of study participants (n=32) and also the time TWOC was done (within 10 days) could be responsible for the higher success rate.

In this study, the mean IPP was 13.04mm, with a higher proportion of grade III IPP in patients with AUR from BPH. The percentages of patients with grade I, grade II and grade III IPP were 28.2%, 24.4% and 47.4% respectively. This is similar to the study by Sharis *et al*. [5] who reported percentages of patients with grade I, grade II and grade III to be 25%, 21.9% and 53.1% respectively. Das *et al*. [25] reported 33.3%, 42.2% and 24.4% as the percentages of patients with grade I, grade II and grade II IPP respectively, which is slightly different from that of this study.

Intravesical prostatic protrusion (IPP) was found to significantly predict the outcome of TWOC in patients with AUR from BPH with an AUC of 0.843 (p=0.001), cut off mark of < 7.4mm, sensitivity of 79.6% and specificity of 83.3%. Patients with IPP less than 7.4mm were more likely to have a successful TWOC. This is similar to the work done by Bhomi *et al*. [23] and Bansal *et al*. [22] who reported that IPP predicted the outcome of TWOC following AUR from BPH with a cut off of 8mm and 9mm respectively. Mariappan *et al*. [15], Tan *et al*. [16], Shanmugasundaram *et al*. [24] and Sharis *et al*. [5] also reported that IPP greater than 10mm (grade III IPP) was a strong predictor of an unsuccessful TWOC in patients with AUR from BPH.

The positive predictive value (PPV) and negative predictive value (NPV) of IPP on the outcome of TWOC at a cut off of 7.4mm were 91.5% and 64.5% respectively. A high positive predictive value for patients at a cut off of < 7.4mm revealed a high probability of a successful TWOC when IPP is <7.4mm. Thus, IPP was a good tool for predicting the success of TWOC in patients with AUR from BPH. The limitation of this study is that compliance with tamsulosin therapy could not be guaranteed as patients were not admitted for the study. The author minimized this by giving the patient a one week supply of tamsulosin and also properly educated the patient on the importance of tamsulosin and how to take it.

## Conclusion

This study revealed that IPP significantly predicted the outcome of TWOC in patients with AUR from BPH. Patients with IPP less than 7.4mm are more likely to have a successful trial without catheter following relief of AUR from BPH, while those with IPP ≥ 7.4mm should be considered for early surgical intervention rather than TWOC. Further research should be directed at multicenter study and meta-analysis of the predictive value of IPP on outcome of TWOC in BPH patients with AUR.

### What is known about this topic

- Acute urinary retention is the sudden inability to pass urine requiring catheterization, it has a significant impact on patients´ health-related quality of life and it is of substantial economic burden;
- A successful TWOC not only delays surgical intervention for BPH, but may also avoid it;
- Intravesical prostatic protrusion is a useful predictor in evaluating the success of a voiding trial following AUR.

### What this study adds

- Intravesical prostatic protrusion significantly predicted the outcome of TWOC in BPH patients presenting with AUR with a high PPV;
- Patients with IPP ≥7.4mm should be considered for early intervention rather than TWOC.
